# Obesity as an Emerging Risk Factor for Iron Deficiency

**DOI:** 10.3390/nu6093587

**Published:** 2014-09-11

**Authors:** Elmar Aigner, Alexandra Feldman, Christian Datz

**Affiliations:** 1 First Department of Medicine, Paracelsus Medical University, Müllner Hauptstrasse 48, 5020 Salzburg, Austria; E-Mail: a.feldman@salk.at; 2 Obesity Research Unit, Paracelsus Medical University, Müllner Hauptstrasse 48, 5020 Salzburg, Austria; 3 Department of Internal Medicine, General Hospital, Paracelsusstrasse 37, 5110 Oberndorf, Austria

**Keywords:** iron deficiency, obesity, insulin resistance, hepcidin

## Abstract

Iron homeostasis is affected by obesity and obesity-related insulin resistance in a many-facetted fashion. On one hand, iron deficiency and anemia are frequent findings in subjects with progressed stages of obesity. This phenomenon has been well studied in obese adolescents, women and subjects undergoing bariatric surgery. On the other hand, hyperferritinemia with normal or mildly elevated transferrin saturation is observed in approximately one-third of patients with metabolic syndrome (MetS) or nonalcoholic fatty liver disease (NAFLD). This constellation has been named the “dysmetabolic iron overload syndrome (DIOS)”. Both elevated body iron stores and iron deficiency are detrimental to health and to the course of obesity-related conditions. Iron deficiency and anemia may impair mitochondrial and cellular energy homeostasis and further increase inactivity and fatigue of obese subjects. Obesity-associated inflammation is tightly linked to iron deficiency and involves impaired duodenal iron absorption associated with low expression of duodenal ferroportin (FPN) along with elevated hepcidin concentrations. This review summarizes the current understanding of the dysregulation of iron homeostasis in obesity.

## 1. Introduction

Both iron deficiency (ID) and obesity are global epidemics affecting billions with regional disparities [1]. It has become clear that iron deficiency and obesity do not merely represent the coincidence of two frequent conditions but are molecularly linked and mutually affect each other [2]. While obesity has become a socioeconomic burden in industrialized countries over the last century, the prevalence is currently also increasing in developing countries with the spread of energy-dense food compounds and a sedentary lifestyle [3]. The most important sequelae of obesity include cardiovascular diseases, type 2 diabetes (T2DM), and an increased rate of several cancers [4,5]. The interaction of iron homeostasis with obesity represents a Janus-faced clinical condition. On the one hand, obesity may promote iron deficiency by inhibition of dietary iron uptake from the duodenum. On the other hand, a condition termed “dysmetabolic iron overload syndrome (DIOS)” has become the most frequent differential diagnosis for elevated ferritin concentrations, affecting approximately one-third of subjects with nonalcoholic fatty liver disease (NAFLD) or metabolic syndrome (MetS). DIOS is characterized by increased serum ferritin concentrations with normal or mildly elevated transferrin saturation in subjects with various components of MetS or NAFLD. True iron overload is rarely found in liver biopsy, and liver iron only weakly correlates with serum ferritin concentrations. It is associated with more severe manifestations and outcomes. From a therapeutic perspective, iron removal via phlebotomy has been established to convey additional benefit [2]. From a clinical point of view, both conditions are relevant and require adequate diagnostic work-up and treatment. This review is mainly focused on iron deficiency (ID) in obesity and aspects of DIOS are only mentioned where relevant to the main topic. Details of the clinical relevance and mechanisms of DIOS have been reviewed elsewhere [2,6].

## 2. The Pathophysiology of Obesity

This paragraph outlines important pathomechanisms of obesity and related conditions, particularly those pertaining to the dysregulation of iron homeostasis.

Although an increase in adipocyte mass and number is the morphological hallmark of obesity, functional changes of obese compared to lean adipose tissue (AT) are of paramount importance. Obese AT is characterized by macrophage infiltration and local production of pro-inflammatory cytokines such as interleukin-1 (IL-1), IL-6 and tumor-necrosis-factor-α (TNF-α) sustaining a low-grade systemic inflammatory milieu [7]. In addition, the expression of hormone-like peptides, referred to as adipokines (analogous to cytokines, derived from adipocytes) is markedly changed as AT progresses from the lean to the obese phenotype [8]. Adiponectin serves as an anti-inflammatory and insulin-sensitizing agent, and its concentrations are decreased in obesity and insulin resistance (IR) [9]. Leptin most likely indicates satiety and fullness of energy stores under physiological conditions, but obesity is characterized by hyperleptinemia and hypothalamic leptin resistance [10]. Resistin is closely linked to AT inflammation, and most likely also to cardiovascular diseases and insulin resistance [11]. Adipocyte fatty acid binding protein (A-FABP) expressed in mature adipocytes and activated macrophages is positively associated with parameters of adiposity, IR and metabolic syndrome. Rodents lacking A-FABP are protected from several adverse consequences of obesity [12]. IR renders adipocytes with increased lipolytic activity leading to an increased flux of free fatty acids from AT to the liver or muscle [13]. In summary, inflammatory cytokines, adipokines and free fatty acids collaborate in mediating adverse health consequences from the AT to other organs.

The liver, which is also the key regulator of iron homeostasis, in obesity and IR is characterized by lipid accumulation called nonalcoholic fatty liver disease (NAFLD). Liver steatosis is mostly benign but the severe form of NAFLD, nonalcoholic steatohepatitis (NASH), is characterized by inflammation and fibrosis and may potentially progress to cirrhosis, end-stage liver disease or hepatocellular carcinoma in a minority of patients [14]. Increased insulin secretion from the pancreas is frequently observed in obesity to compensate for developing tissue IR but, ultimately, secretory failure will lead to overt type 2 diabetes.

## 3. The Physiology of Iron Metabolism

Many aspects of the physiological regulation of human iron homeostasis have been elucidated over the past decade [15]. Iron is absorbed as Fe^2+^ in the proximal duodenum by the divalent metal transporter 1 (DMT1) [16]. Following its transfer through the duodenal basolateral membrane facilitated by the iron exporter ferroportin (FPN) [17], iron undergoes oxidation by the membrane-bound copper containing ferroxidase hephaestin [18] before being incorporated into transferrin for further transport into circulation. Although heme constitutes an important source of iron from the diet and also supplies iron for cellular iron requirements via re-utilization, the mechanism for enteral heme uptake has not yet been identified [19]. Most cells acquire iron via the uptake of transferrin-bound iron (Fe^3+^) by the transferrin receptor (TfR1). Iron is mainly required for heme biosynthesis in the erythropoietic bone marrow and other heme containing enzymes (e.g., cytochromes), whereas excess iron is stored in the liver hepatocytes. Iron is exported from hepatocytes, macrophages and all other mammalian cells via FPN, which has thus far been identified as the only iron exporter [15].

Systemic iron homeostasis is maintained in a hormone-like negative feedback mechanism by the 25-amino acid peptide hormone hepcidin (hepatic bactericidal protein) [20]. Hepcidin is secreted from hepatocytes in response to iron overload, inflammation, hypoxia or anemia. Hepcidin exerts its regulatory functions on iron homeostasis via binding to FPN, thereby leading to FPN phosphorylation, degradation and consequently to blockage of cellular iron export which induces a decrease in serum iron [21]. Although in quantitative terms the liver is the main source of circulating hepcidin, macrophages, pancreatic islet cells and adipose tissue can also express hepcidin [22,23].

## 4. The Iron Phenotype of Obesity

**Figure 1:**
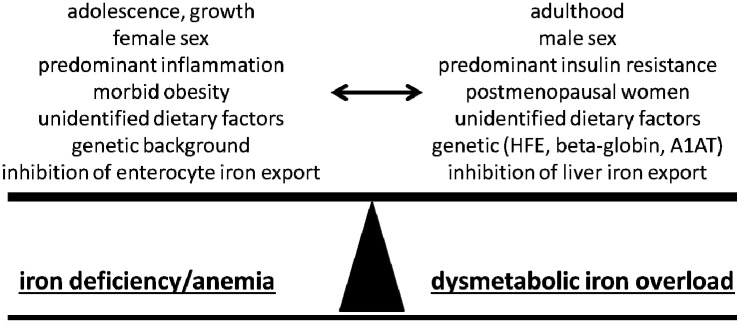
Summary of potential stimuli and characteristics which may affect the manifestation of the iron phenotype in obesity. Both iron deficiency and the dysmetabolic iron overload syndrome represent well-studied manifestations of disordered iron homeostasis. The net balance of underlying, frequently competing stimuli will likely define the iron phenotype in an individual patient.

It appears counterintuitive that obesity as a condition of calorie and nutrient excess is associated with ID. However, the profound changes in energy homeostasis in AT, the liver and also other organs involved are closely linked to distinct changes of iron homeostasis. Iron dysregulation in obesity may, in the manner of the Roman god Janus, present with two sides facing opposite directions. Data from pathophysiological studies strongly suggest that these two distinct clinical problems are in fact manifestations of the same underlying mechanisms with obesity-related iron deficiency on one side and DIOS on the other. It appears reasonable to assume that the observed iron phenotype finally results from the net balance of often competing stimuli. Figure 1 depicts a summary of these stimuli which appear related to a distinct phenotype of iron homeostasis in obese and/or insulin-resistant subjects.

## 5. Clinical Data on Obesity-Related Iron Deficiency—Adolescence

Lower concentrations of serum iron with increasing BMI were observed several decades ago and confirmed in subsequent investigations [24,25,26]. An analysis from the NHANES population found the risk for ID, defined as low transferrin saturation and low serum ferritin, to be twice as high in overweight adolescents compared to normal weight adolescents [27], with similar results reported from an Israeli population [28]. Comparable results were documented in Iranian and Chinese investigations [29,30]. A further US study found a strong link between ID and BMI across all races, ages and amounts of dietary intake [31]. These studies unequivocally demonstrated lower serum iron availability with increasing AT mass in adolescents. Furthermore, iron uptake from the duodenum is limited in obese compared to normal weight children [32]. Conversely, a study in mostly lean or only mildly overweight adolescents without severely obese subjects concluded that higher serum ferritin concentrations and transferrin saturation correlated with increasing BMI quartiles—a finding reminiscent of associations commonly reported in adult populations [33].

## 6. Adulthood

In adults, several analyses demonstrated lower serum iron concentrations with higher BMI, particularly in women. However, results appear much more complex in adults compared to adolescents. Hence, one study reported lower serum iron concentrations in overweight women but no differences in males [34]. No differences in serum iron between obese and normal weight controls were documented in another investigation [35]. In morbid obesity and bariatric surgery cohorts, ID has been identified as a typical and frequent condition as detailed below [36].

The association between adult obesity and low iron stores or anemia has been evaluated in a recent meta-analysis of all controlled studies [37]. Although ID appears as a typical finding in severe obesity, the review concluded that most studies demonstrated higher hemoglobin and ferritin concentrations in obese subjects compared to normal weight adults. However, serum iron and transferrin saturation decrease as BMI increases. This has mainly been attributed to a growing effect of obesity-related inflammation with increasing grades of obesity.

In conclusion, considering adolescence and adulthood, one may assume that the increased iron demand, owing to physical growth, increased fat mass or blood volume [27,38], in conjunction with diminished iron uptake in adolescent obesity, may explain the clear-cut association of obesity with iron deficiency in this cohort [28]. This association is lost during later stages of life, e.g., postmenopausal women or insulin-resistant men, and factors favoring iron accumulation may even prevail. However, it remains relevant in pronounced stages of obesity such as bariatric surgery cohorts or women [39]. In these cohorts, menstrual iron loss or pronounced AT inflammation may tip the balance towards ID, whereas this appears to be a rare finding in men as summarized in Figure 1.

## 7. Response of Iron Parameters to Weight Reduction

Important data has been obtained from the study of changes in iron homeostasis in response to weight loss. Gong *et al.* reported an improvement of iron status with unchanged serum ferritin concentrations and an increase in transferrin saturation after the intervention along with improvement of inflammatory markers [40]. Similarly, Amato *et al.* observed a decrease of serum hepcidin along with increased iron absorption after a six-month weight-loss program in children [41]. However, no changes in iron status were observed in another study, though iron parameters were generally normal at baseline [42]. A very low-calorie diet even induced a further decrease in iron stores in obese children; however, the intervention performed does not represent a healthy, recommendable mode of weight loss [43]. Insightful data have been derived from the observation of improvement of functional iron status as indicated by increased transferrin saturation and decreased serum hepcidin concentration in response to weight loss due to restrictive bariatric surgery [44]. Largely, the prevailing evidence suggests that a healthy mode of weight loss in obese subjects is accompanied by an improvement of inflammatory markers along with re-established dietary iron absorption and serum iron concentrations as an indication for re-establishment of physiological iron homeostasis.

## 8. Relevance of ID to the Course of Obesity

Data is relatively scarce with regard to the relevance of ID to the course of obesity. However, several commonsense assumptions are indirectly supported by observational studies. ID and anemia may lead to fatigue and thereby to an additional decrease in physical activity, further aggravating weight gain [45]. Additionally, ID may impair mitochondrial respiratory chain activity, thereby limiting exercise capacity and augmenting insulin resistance [26]. Supporting this line of evidence, an improvement of several metabolic parameters was demonstrated with the correction of ID [46].

## 9. Bariatric Surgery and Iron Deficiency

The European guidelines on bariatric surgery state that bariatric surgery is indicated *inter alia* in patients with BMI ≥ 40 kg/m^2^ or ≥35 kg/m^2^ with obesity-related co-morbidities such as metabolic complications, cardiorespiratory or joint disease [47]. Bariatric surgeries are classified as restrictive or malabsorptive. Roux-en Y gastric bypass (RYGB), as well as biliopancreatic diversion with or without duodenal switch (BPD and BPD-DS), are combined restrictive and malabsoprtive procedures. Restrictive surgeries include sleeve gastrectomy (SG), adjustable gastric banding (AGB), and vertical banded gastroplasty. The most frequently performed methods are RYGB, AGB and SG [48]. Recently, duodenal-jejunal bypass sleeve (Endobarrier^®^) has been introduced as a novel, endoscopically applied treatment option for obesity and type 2 diabetes [49]. To date, no investigations have been performed on the changes of iron homeostasis in these subjects.

Study results on the micronutritional status in patients prior to bariatric surgery vary widely and show that low iron and low ferritin serum concentrations are observed in 30% to 40% and 6% to 9% respectively [50,51,52]. An early study by Harju *et al.* indicated that a low ferritin concentration before surgery may be associated with higher complication rates [53]. However, no positive effect of perioperative intravenous iron on the postoperative clinical outcome has been found [54,55]. In representative investigations, the prevalence of iron deficiency one year after RYGB, AGB and SG was estimated to be 20%, 14% and 15%, respectively [51,56,57]. Several studies compared different bariatric methods regarding the micronutrient status after surgery. In a recent study comparing RYGB and SG, no significant difference occurred in relation to iron deficiency or anemia in a mean follow-up period of four years with similar results obtained by Gehrer *et al.* [58,59]. In line with this, no significant difference of iron deficiency was found by Coupaye when comparing AGB and RYGB in a one-year prospective study [57]. As mentioned above, the restrictive bariatric interventions AGB and sleeve gastrectomy improved functional iron status as indicated by a decrease in hepcidin and an increase in transferrin saturation [44]. Nonetheless, improvement in functional iron status was reported along with weight reduction after restrictive procedures, whereas after malabsorptive procedures, worsening of ID or at least persistence is frequently observed [60,61].

According to the practice guidelines of the American Society for Metabolic and Bariatric Surgery, iron status should be assessed at every follow-up visit after bariatric surgery. In case of ID, a daily iron dosage of 150 to 200 mg should be provided via supplements. Further, it is recommended for patients with RYGB to receive 45 to 60 mg of iron daily during the first six months [62]. However, according to a recent study by Gesquiere, oral supplements are insufficient to overcome impaired absorption after RYGB and it is therefore advised to treat severe ID by parenteral substitution [63].

In summary, bariatric surgery patients are at high risk of suffering from ID before surgery. Malabsorptive strategies may further lead to ID despite adaptive up-regulation of jejunal iron transporters, most likely due to the significant loss of absorptive surface [64,65]. After restrictive surgery, improvement of functional iron status is observed in a significant proportion of patients, which appears to relate to weight loss and consecutive improvement of AT inflammation [39,66].

## 10. Mechanisms Underlying Iron Deficiency in Obesity

The central finding of studies examining iron homeostasis in obese subjects represents an impaired ability of duodenal iron absorption. Markedly lower isotope-labeled iron absorption in obese compared to overweight and normal weight subjects with or without ascorbic acid was found [67]. Similar observations were reported in obese men and children from subsequent investigations [65,68]. Thus, decreased dietary iron uptake due to lower enterocyte iron absorption can be regarded as the pathophysiological hallmark of iron dysregulation in obesity. 

**Figure 2:**
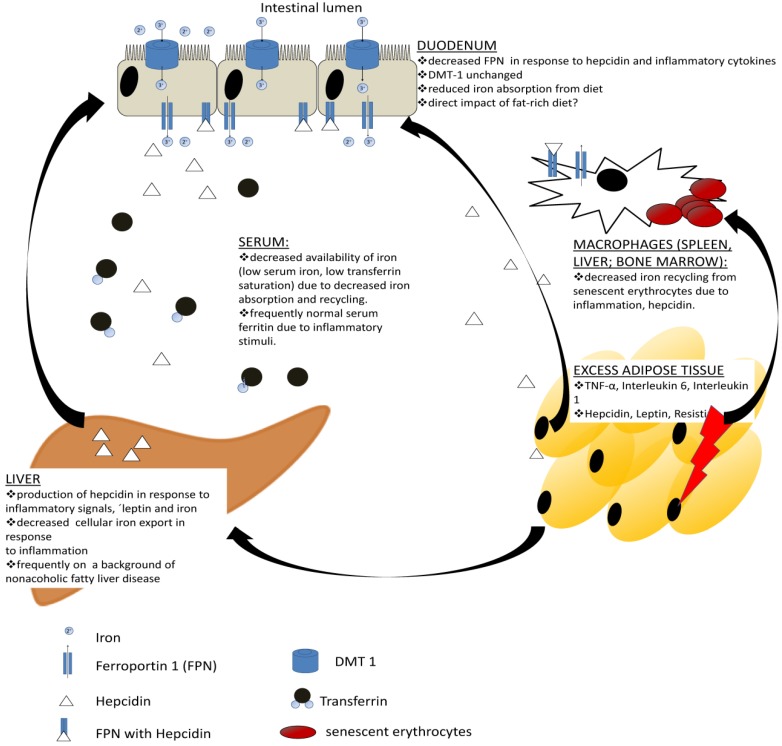
Current understanding of molecular links between obesity and iron deficiency. Obese adipose tissue is characterized by an increased production of several pro-inflammatory cytokines and adipokines as opposed to healthy lean adipose tissue. These may directly impact iron absorption from the enterocyte. Additionally, pro-inflammatory cytokines such as interleukin-1 and -6 represent potent inducers of hepcidin production in the liver, which may further impair iron absorption. Both cytokines and hepcidin lead to iron retention in spleen, liver or bone marrow macrophages, thereby lowering serum iron concentrations and iron availability for erythropoiesis.

Since the liver-derived peptide hormone hepcidin represents the master regulator of iron homeostasis, its role has been investigated in the context of obesity. Elevated serum hepcidin concentrations have been reported in severe obesity with associated anemia [22,69,70], but also in studies investigating mechanisms underlying the DIOS [71,72]. As decreased enteral iron absorption and elevated hepcidin expression are found in both obesity-related iron deficiency and DIOS, we suggest that these conditions represent different manifestations of the same underlying pathophysiological process [67,73]. While elevated hepcidin in DIOS is related to increasing iron stores, in severe obesity with iron deficiency it appears primarily linked to inflammatory markers [71,74]. Irrespective of the underlying cause, elevated hepcidin concentrations explain lower duodenal FPN expression and diminished dietary iron absorption. Although the liver is traditionally regarded as the main source of hepcidin, expression of hepcidin in the adipose tissue from anemic, iron-poor, morbidly obese subjects has been demonstrated which is absent in lean adipose tissue [22]. As AT mass increases, the contribution of AT-derived hepcidin may become substantial, although the relevance of AT hepcidin has been questioned by a recent investigation due to very low amount of hepcidin mRNA compared to expression in the liver and lack of evidence of a substantial release from AT [75]. It appears plausible, however, that AT-derived cytokines such as IL-6 and IL-1 function as potent inducers of hepcidin expression in the liver also in obesity [76]. This conclusion is further supported by the aforementioned clinical data, where an improvement of functional iron status has been observed in response to weight loss [44]. The current understanding of mechanisms underlying iron deficiency in obesity is summarized in Figure 2. Furthermore, one may speculate that pro-inflammatory cytokines interfere with erythropoietin production and also blunt the response of erythroid precursors to erythropoietin which is a well-recognized mechanism in the development of anemia of chronic disease and may thus also contribute to anemia in obese subjects [77]. Rodent models suggest that a fat-rich diet may inhibit duodenal iron uptake via a hepcidin-independent mechanism [78]. Moreover, the interaction between copper availability and iron homeostasis represents a potential link between dietary factors and iron uptake as low copper may lead to reduced ferroxidase activity necessary for iron export from enterocytes, macrophages and hepatocytes [79,80].

## 11. Conclusions

Abnormal parameters of iron status indicating iron deficiency or overload are frequent findings in overweight and obese subjects. Iron deficiency represents a particular clinical problem during adolescence when iron requirements are increased, and in morbid obesity during adulthood. Impaired functional iron status is mainly linked to adipose tissue inflammation and increased expression of the systemic iron regulatory protein hepcidin. Cytokines such as TNF-α, IL-1 and IL-6 along with adipokines (leptin, resistin) or hepcidin may represent signals from obese, inflamed AT facilitating changes in physiological iron homeostasis. Owing to its underlying mechanism of impaired iron absorption via the gut, treatment of iron deficiency by oral supplementation is frequently insufficient, and parenteral substitution is thus necessary, particularly in bariatric surgery patients. As both iron deficiency and overload may have detrimental effects on the course of obesity-related conditions, diligent screening and treatment of both is warranted.
